# Supplementary material for: Protein polyglutamylation catalyzed by the bacterial calmodulin-dependent pseudokinase SidJ
Source: eLife. 2019 Nov 4;8:e51162. doi: 10.7554/eLife.51162 (PMC6858067; doi:10.7554/eLife.51162)
Supplement: Supplementary file 1. [file elife-51162-supp1.docx]

**Supplementary File 1: Key Resources Table.**

| **Reagent type (species) or resource** | **Designation** | **Source or reference** | **Identifiers** | **Additional information** |
| --- | --- | --- | --- | --- |
| gene (*Legionella pneumophila*) | SidJ: lpg2155 | NCBI | NCBI Gene ID: 19833721 | Cloned from genomic DNA. See materials and methods section. |
| gene (*Legionella pneumophila*) | SdeA: lpg2157 | NCBI | NCBI Gene ID:19833723 | Cloned from pEGFP-SdeA plasmid from PMID: 27049943. |
| strain, strain background (*Escherichia coli*) | Rosetta(DE3) | Sigma -Aldrich | Cat. #: 70954 | Protein purification strain |
| strain, strain background (*Escherichia coli*) | Top10 | Life Technologies | Cat. #: C4040 | DNA propagation and cloning strain |
| strain, strain background  *(Legionella Pneumophila)* | LP02 | PMID: 8382332 |  | *Legionella* strain |
| genetic reagent (*Legionella pneumophila*) | LP02*ΔsidJ* | This paper |  | *Legionella* deletion strain of SidJ. See materials and methods for development. |
| genetic reagent (*Legionella pneumophila*) | LP02*Δ4sidE* | PMID: 27049943 |  | *Legionella* deletion strain of SidE family PR-Ub ligases |
| cell line (*Homo sapiens*) | HEK293T | ATTC | CRL-3216 | Tested negative for mycoplasma |
| antibody | Anti-FLAG (mouse, monoclonal) | Sigma-Aldrich | Cat. #: F1804 | (1:8,000) |
| recombinant DNA reagent | pET21-Ub | PMID: 25006264 |  | Protein purification of Ubiquitin |
| recombinant DNA reagent | pET28a-6xHis-Sumo-Calmodulin2 | This paper |  | For protein purification. See materials and methods for details. |
| recombinant DNA reagent | pET28a-6xHis-Sumo-SdeA (211-1152) | This paper |  | SdeA "Core" truncation plasmid used for protein purification. See materials and methods for details. |
| recombinant DNA reagent | pET28a-6xHis-Sumo-SdeA (211-910) | This paper |  | SdeA truncation plasmid used for protein purification. See materials and methods for details. |
| recombinant DNA reagent | pET28a-6xHis-Sumo-SdeA (211-758) | This paper |  | SdeA truncation plasmid used for protein purification. See materials and methods for details. |
| recombinant DNA reagent | pET28a-6xHis-Sumo-SidJ (89-853) | This paper |  | SidJ truncation plasmid used for crystallization and protein purification. See materials and methods for details. |
| recombinant DNA reagent | pET28a-6xHis-Sumo-SidJ K367A (89-853) | This paper |  | SidJ active site point mutation plasmid used for protein purification. See materials and methods for details. |
| recombinant DNA reagent | pET28a-6xHis-Sumo-SidJ E381 (89-853) | This paper |  | SidJ active site point mutation plasmid used for protein purification. See materials and methods for details. |
| recombinant DNA reagent | pET28a-6xHis-Sumo-SidJ R352A (89-853) | This paper |  | SidJ active site point mutation plasmid used for protein purification. See materials and methods for details. |
| recombinant DNA reagent | pET28a-6xHis-Sumo-SidJ D489A (89-853) | This paper |  | SidJ active site point mutation plasmid used for protein purification. See materials and methods for details. |
| recombinant DNA reagent | pET28a-6xHis-Sumo-SidJ N534A (89-853) | This paper |  | SidJ active site point mutation plasmid used for protein purification. See materials and methods for details. |
| recombinant DNA reagent | pET28a-6xHis-Sumo-SidJ D542A (89-853) | This paper |  | SidJ active site point mutation plasmid used for protein purification. See materials and methods for details. |
| recombinant DNA reagent | pET28a-6xHis-Sumo-SidJ D545A (89-853) | This paper |  | SidJ active site point mutation plasmid used for protein purification. See materials and methods for details. |
| recombinant DNA reagent | pET28a-6xHis-Sumo-SidJ H492A (89-853) | This paper |  | SidJ nucleotide binding pocket point mutant plasmid used for protein purification. See materials and methods for details. |
| recombinant DNA reagent | pET28a-6xHis-Sumo-SidJ Y506A (89-853) | This paper |  | SidJ nucleotide binding pocket point mutant plasmid used for protein purification. See materials and methods for details. |
| recombinant DNA reagent | pET28a-6xHis-Sumo-SidJ IQ mutant (89-853) | This paper |  | SidJ IQ motif mutant plasmid used for protein purification. See materials and methods for details. |
| recombinant DNA reagent | pET28a-6xHis-Sumo-SidJ (110-853) | This paper |  | SidJ truncation plasmid for protein purification. See materials and methods for details. |
| recombinant DNA reagent | pmCherry-SidJ | This paper |  | Full length gene used for mammalian cell transfection. |
| recombinant DNA reagent | pEGFP-SdeA | PMID: 27049943 |  | Full length gene used for mammalian cell transfection. |
| recombinant DNA reagent | pcDNA3.1(+)-4xFlag-Rab33b | PMID: 27049943 |  | Full length gene used for mammalian cell transfection. |
| recombinant DNA reagent | pZL507-SidJ | This paper |  | For expression of SidJ in *Legionella*. |
| recombinant DNA reagent | pZL507-SidJ D542A | This paper |  | For expression of SidJ mutant in *Legionella*. |
| recombinant DNA reagent | pSR47s-SidJ_Knockout | This paper |  | For deletion of SidJ from *Legionella*. Original plasmid from: PMID 20333253 |
| recombinant DNA reagent | pCMV-FcγRII | PMID: 27049943 |  | Transfection for *Legionella* infection |
| Sequence Based Reagent | Sidj_89_BamHI_F | This paper | PCR Primers | agaggatccggccccaaagtaacac |
| Sequence Based Reagent | SidJ_853_SalI_R | This paper | PCR Primers | cgcgtcgactcatgacttgcgtgcttctcgaagttt |
| Sequence Based Reagent | Calmodulin2_BamHI_F | This paper | PCR Primers | gacggatccatggctgaccaactgactgaagagc |
| Sequence Based Reagent | Calmodulin2_XhoI_R | This paper | PCR Primers | cgtctcgagtcactttgctgtcatcatttgtacaaactc |
| Sequence Based Reagent | SdeA_211_BamHI_F | This paper | PCR Primers | gacggatccatgtctgttaaaccc |
| Sequence Based Reagent | SdeA_1152_XhoI_R | This paper | PCR Primers | gacctcgagttatccaacgtgcatccg |
| Sequence Based Reagent | SidJ_Upstream_SalI_Fwd | This paper | PCR Primers for LP02 KO | gacgtcgacTTTAATTTAAACAAAAGGTGGTTATTGTGCCC |
| Sequence Based Reagent | SidJ_Upstream_BamH1_Fwd | This paper | PCR Primers for LP02 KO | gacggatccATCAACACCAAAAAAATCAAGTACTTTCTTTATGAAACC |
| Sequence Based Reagent | SidJ_Downstream_BamH1 | This paper | PCR Primers for LP02 KO | gacggatccTTCTGAAAAGCCGGAATCCGAACG |
| Sequence Based Reagent | SidJ_Downstream_SacI | This paper | PCR Primers for LP02 KO | gacgagctcCTCAGTTGAGCTTTACGTGCTTCTTCTATC |
| peptide, recombinant protein | MBP (Myelin Basic Protein) Bovine origin | Thermo Fisher | Cat. #: 13228010 | Phosphorylation substrate biochemical reactions |
| chemical compound, drug | Adenosine 5' Triphosphate (ATP) | Sigma-Aldrich | Cat. #: A2383-5G | Biochemical reactions |
| chemical compound, drug | β-Nicotinamide adenine dinucleotide sodium salt (NAD) | Sigma-Aldrich | Cat. #: N0632 | Biochemical reactions |
| chemical compound, drug | [γ-^32^P]ATP | Perkin Elmer | Cat. #: BLU002Z250UC | Biochemical reactions |
| chemical compound, drug | [α-^32^P]ATP | Perkin Elmer | Cat. #: BLU003X250UC | Biochemical reactions |
| chemical compound, drug | L-[U-^14^C]Glu | Perkin Elmer | Cat. #: NEC290E050UC | Biochemical reactions |
